# Crape myrtle bark scale *Acanthococcus lagerstroemiae* (Coccidae: Eriococcidae) infestation seasonally alters the insect biodiversity on crape myrtle trees

**DOI:** 10.1007/s00442-025-05792-3

**Published:** 2025-09-10

**Authors:** Elijah P. Carroll, David W. Held, Nash E. Turley, Selina Bruckner

**Affiliations:** 1 Department of Entomology and Plant Pathology, Auburn University, 301 Funchess Hall, Auburn, AL 36849 USA; 2 Department of Entomology, Pennsylvania State University, 501 Agricultural Sciences and Industries Building, State College, PA 16803 USA

**Keywords:** Felt scale, Urban forestry, Biotic facilitation, Invasive species, Seasonal turnover

## Abstract

Understanding changes to local communities brought about by biological invasions is important for conserving biodiversity and maintaining environmental stability. Scale insects (Hemiptera: Coccoidea) are a diverse group of insects well known for their invasion potential and ability to modify local abundance of multiple insect groups. Here, we tested how the presence of crape myrtle bark scale (*Acanthococcus lagerstroemiae,* CMBS), an invasive felt scale species, seasonally impacted local insect abundance, biodiversity, and community structure on crape myrtle trees. Our field surveys showed that CMBS-infested trees had seasonal changes to local insect abundance and family-level richness, and inverse Simpson’s diversity relative to non-infested trees. CMBS infestation resulted in a decrease in community evenness on crape myrtle trees. Community compositions of insect visitors were distinct between infested and non-infested trees. CMBS-infested trees had greater seasonal abundances of Coccinellidae, Vespidae, Dolichopodidae, and Muscidae. Two of the families (Coccinellidae, Vespidae) that responded most strongly to CMBS infestation were dominated by non-native species. Our results show that CMBS-infested trees acted as resource hubs for natural enemies and carbohydrate scavengers, resulting in uneven communities shaped by a few highly responsive taxa. The temporal dynamics of these effects support CMBS as an ecological catalyst, reshaping urban insect communities and highlight its potential for facilitating invasion cascades in anthropogenic habitats. These results emphasize the importance of fine-scale temporal monitoring for understanding and mitigating the ecological impacts of introduced scale insects in urban environments.

**Supplementary Information:**

The online version contains supplementary material available at 10.1007/s00442-025-05792-3.

## Introduction

Insect invasions can result in extensive economic and environmental consequences. Despite the increased awareness of the negative impacts posed by insect invasions, the global rate of introductions has been increasing exponentially since the 1950s (Seebens et al. 2017). Scale insects are among the most invasive and ecologically disruptive insect groups with the ability to alter insect communities in both natural and introduced ranges (Ewers 2002; Pellizzari and Germain 2010; Mazzeo et al. 2014). Their obligatory parasitic relationship with plants, small size, cryptic lifestyle, and homologous morphology between certain species makes them one of the most frequently overlooked insect groups during interstate and international trade (Miller et al. 2005; Mazzeo et al. 2014; Frank 2021). It is estimated that scale insects account for 13% of the introduced insect fauna in the USA with an average of one new species introduced to the USA each year (Morales et al. 2016). The horticultural plant trade can act as a major route of dispersion and means of establishment for scale insects into an extended geographic range. For example,~50% of the exotic scale species reported in Italy since 1945 have been found on ornamental plants (Mazzeo et al. 2014).

Once established, scale insects can restructure local arthropod assemblages by supplying persistent, high-energy resources to the environment. They act as a direct trophic input by serving as prey for a range of natural enemies (Gullan and Cook 2007). In introduced ranges where specialist predators are absent, this role is often filled exclusively by generalist predators such as lady beetles, lacewings, spiders, and earwigs (Miller et al. 2004; Logan et al. 2017; Wilson and Frank 2022). Indirectly, scale insects produce honeydew, excreted phloem sugars that act as a carbohydrate resource for many insects including parasitoids, flies, and bees (Tena et al. 2013; Van Rijn et al. 2013; Hung et al. 2015; Meiners et al. 2017). Honeydew can supplement nectar, especially during floral dearth periods, and is often more abundant and easier to access than floral nectaries (Calvo-Agudo et al. 2022). It also has differences in sugar composition and plant-associated metabolites, which contribute to a greater variability in nutritional suitability for insects relative to nectar (Wäckers et al. 2008). Thus, honeydew may disproportionately benefit opportunistic species such as generalist scavengers and facilitate community structures centralized around generalist carbohydrate foraging taxa (Beggs 2001; Styrsky and Eubanks 2007; Vanek and Potter 2010; Zhou et al. 2012, 2017; Gardner-Gee and Beggs 2013).

The impact of invasive species introductions is well known to vary seasonally (Alaya et al. 2007; Coulter et al. 2018; White and Hastings 2020). For scale insects, honeydew production and population density can change throughout the year, while local environmental conditions (e.g., floral availability, prey abundance) also shift over time. These temporal dynamics may amplify or dampen the influence of scale insects on recipient communities depending on the season. While many studies have documented scale insect-driven enrichment of insect communities, few have investigated how the strength or direction of these effects changes across seasons. To date, only one study has explicitly evaluated seasonal shifts in insect biodiversity in response to an introduced scale species (Petrakis et al. 2011), underscoring the need for more temporally resolved assessments of invasion effects.

Crape myrtle (*Lagerstroemia indica* L.), a deciduous woody plant native to China and Korea, was introduced into the USA in 1747 for ornamental uses (Dirr 1990). It has become a dominant plant component in urban and suburban landscapes in eastern North America because it grows well in the often poor soil conditions in urban environments and has the desirable aesthetics of abundant, long-lived blooms (Chappell et al. 2012). This tree is rarely utilized by native insect herbivores, and its primary herbivores are introduced species (Clem and Held 2018). These include two honeydew-producing hemipterans: crape myrtle aphids (*Sarucallis kahawaluokalani*) and crape myrtle bark scale (*A. lagerstroemiae,* CMBS), which occur on crape myrtle in their native range and have been reunited with crape myrtle in North America. CMBS, an invasive felt scale, first reported in Dallas Co, TX in 2004 (Wang et al. 2016), has escaped the specialist natural enemies that regulate its population in its native range (Wang et al. 2016). The environments where crape myrtles are planted may also contribute to a reduction in habitat specialists (Parsons and Frank 2019). The introduction of CMBS into North America offers a unique opportunity to examine how an invasive herbivore can restructure an ecologically simplified arthropod community.

Because both crape myrtle trees and their associated herbivore, crape myrtle bark scale (CMBS), have not co-evolved with native insect communities in the USA, they lack specific ecological interactions in their introduced range. As a result, we expect that CMBS infestation will generate a novel insect community structured primarily around generalist taxa. Using a replicated field design, we examined how CMBS infestation influences the temporal dynamics of insect biodiversity on crape myrtle trees. We also compare seasonal indicator species of crape myrtle trees infested and non-infested with CMBS. An indicator taxon is one whose presence is both frequent and relatively exclusive to a given condition. Tracking which taxa are strongly indicative of CMBS-infested trees in each month therefore reveals which seasonal associations between CMBS and insect taxa most strongly restructure the insect community on crape myrtle trees. To do this, we collected data over an 8-month period on the abundance and identity of insect taxa visiting potted crape myrtle trees, both infested and non-infested. We hypothesize that (1) CMBS infestation increases the availability of trophic resources, leading to elevated insect abundance and richness, and (2) because CMBS has escaped its co-evolutionary history with specialized natural enemies and mutualists, interactions in the introduced range will be dominated by generalist and opportunistic taxa, resulting in reduced community evenness and altered community composition. From these hypotheses, we predict that (3) infested and non-infested trees will support compositionally distinct insect communities, and (4) the months in which taxa identified as indicators of CMBS infestation surge in abundance will coincide with seasonal treatment-level differences in insect biodiversity metrics between infested and non-infested trees.

## Materials and methods

### Source of crape myrtle trees and CMBS

Crape myrtle trees (“White Natchez” cultivar) in 51.75 L (50 cm diameter, 42 cm height) plastic pots growing in soilless-bark media were used for the study. These trees were 1.65–2.25 m tall and obtained from a local nursery in Alabama in spring 2020 with no history of insecticide applications (personal inquiry, Moore and Davis Nursey, LLC). Trees were kept in a screenhouse before and between experimental trials, while control trees were held separately on a container pad lined with mesh to prevent infestation. All flowers were removed from the trees prior to surveying each month to prevent bias that may have resulted from insect floral visitation.

Crape myrtle bark scale used to infest the potted trees prior to the experiment were from clippings taken from infested trees established in a shade house on the Auburn University campus. Three clippings, ~ 15 cm long, in plastic floral tubes were tethered to the main trunk of the experimental crape myrtles using Parafilm^®^. Clippings were replaced every 4 days until mature females were visible on the trees used for testing. CMBS populations consist of overlapping generations of mixed life stages from first instar crawlers to mature females. Throughout the study, all infested trees had at minimum ten gravid females per tree per observation month.

### Experimental design

Our treatment groups included crape myrtle trees infested with CMBS and non-infested crape myrtle trees. Twenty potted crape myrtle trees, 10 infested and 10 non-infested with CMBS, were split between two sites in Auburn, Alabama (USA) for observations in summer and fall of 2020 and spring 2021. Trees, infested and non-infested, were paired with 3 m between them and 15 m between each replicate pair. Trees were placed on each site 48 h prior to data collection.

The sites used in this study were near roads and had low to moderate levels of impervious surface. Both sites consisted of little to no overstory layer and understory consisting of mowed turfgrass. One site was on~1.6 hectare (ha) greenspace with sparsely scattered overstory trees consisting of walnut (*Juglans* spp.). This site was adjacent to a 6.72 ha forest patch with two man-made ponds. The other site was on 1.1 ha of greenspace with no overstory.

### Visitation of insects to CMBS-infested crape myrtle trees

Pairs of infested and non-infested trees were surveyed between the months of June–October 2020 and March–May 2021. Insect surveys were conducted on two consecutive days for each month at 0600, 0900, 1200, and 1500 h, apart from April and May when 1 day of data collection was performed (Table S1). Five-minute snapshot surveys were conducted by two observers in the same replicate, each assigned to a tree at random. Insect visitors on the whole tree were counted and identified to family level. Individuals that could not be definitively identified to family in the field were collected into labeled bags and transported to the laboratory for identification using a dichotomous key (Triplehorn et al. 2005). After observation periods, videos of insect behaviors (e.g., honeydew feeding, competitive interactions) were recorded for common taxa (Supplementary). Ants were identified to species level and recorded as a plus/minus for each observation month overall for infested and non-infested trees. Abundances of ants were not recorded because of the difficulty of counting abundant ants during the summer months. Voucher specimens were placed into Auburn University’s Museum of Natural History, Auburn, AL.

### Statistical analysis

First, we summed samples from repeated sampling over the 2-day period each month to account for the temporal pseudo-replication. Insect abundance, family-level richness, inverse Simpson’s diversity, and evenness were calculated using the ‘vegan’ package (Oksanen et al. 2013). Inverse Simpson’s diversity ranges between 1 and the observed richness in the sample. Evenness was calculated as inverse Simpson’s diversity/richness and is related to the relative abundances among species. If all families were equally abundant, evenness would be 1. Four rows of data were removed because they did not have enough families to calculate evenness and diversity metrics, which produced a dataset with 156 samples. Analyses and supporting figures were produced in R version 3.3.2 (R Development Core Team 2016).

To test the effect of infestation, month, and their interaction on changes in insect biodiversity metrics, we used a linear mixed model using the ‘lmer’ function in the ‘lme4’ package (Bates et al. 2014). The model syntax was (*y* ~ infestation status*month, random = 1|site/rep/tree), where infestation was a two-level categorical variable and month was an eight-level categorical variable. The nested random effects in our model account for spatial pseudo-replication by specifying the nesting of trees into paired replicates and replicates into two sites. We obtained P-values using the ‘Anova’ function in the car package (Fox et al. 2012) with type II sums of squares. Following the ANOVA, if a significant effect of treatment, month, or interaction term was detected (*α* < 0.05), means were separated using least-squares means (LS-means) test using ‘emmeans’ in the ‘emmeans’ package (Lenth 2022). In the case of a significant interaction, mean separations were made for comparisons between infested and non-infested trees during each observation month.

We tested the overall effect of infestation on insect community structure by summing the data across months, resulting in one value per individual tree for each treatment (*N* = 20). This was to make the principal component analysis easier to visualize and to account for pseudo-replication from repeated sampling. To test the effects of CMBS infestation on insect community structure, we fit a perMANOVA model using ‘adonis2’ in the ‘vegan’ ’package (Oksanen et al. 2013). The model syntax specified an interaction term for month and treatment. We visualized the community composition results with a principal component analysis.

To identify the overall identifier taxa associated with infested and non-infested trees, we conducted an indicator analysis on the data summed across months using the ‘multipatt’ function in the ‘indicspecies’ package using the indval.g metric for obtaining indicator values (De Cáceres and Jansen 2016). In indicator species analysis, A is the probability that a site belongs to a target site group given that the insect family is present. B is the probability of finding an insect family in the sites belonging to a site group. The indicator value is calculated using the following formula: IV = $\sqrt{A \times B}$ and is equal to 1 when a species was collected in all replicates of a site type and never collected in the other site type. *P-*values were calculated using the Monte Carlo method with 10,000 permutations. To account for multiple comparisons, we applied a false discovery rate (FDR) correction using the ‘p.adjust’ function in the ‘stats’ package in R. To identify taxonomic groups associated with CMBS-infested and non-infested crape myrtle trees for each sampling month, we subset our data by month and conducted indicator species analysis between infested and non-infested trees for each sampling month using the same methods as described above.

## Results

### The influence of CMBS infestation and month on insect biodiversity

Over the course of this study, we recorded 3,767 insects from six orders and 73 families (Table S3). Overall, 67% of the recorded insects were in Diptera, 18% were in Coleoptera, and 12% were in Hymenoptera (excluding Formicidae). The remaining 3% of the recorded insects were divided into Hemiptera (excluding CMBS), Neuroptera, and Psocodea. We collected a total of 58 insect families on infested trees, and 61 families were collected from non-infested trees.

Dolichopodidae was the most abundant taxonomic group and accounted for ~ 47% of all insects recorded during this experiment. Coccinellidae was the second most abundant taxonomic group (~ 16% of all insects recorded), and Phoridae (~ 6% of the recorded insects) were the third most abundant group (Table S2). Thus, the three most abundant families accounted for ~ 70% of all samples. Three ant species recorded on CMBS-infested crape myrtle trees were *Solenopsis invicta, Linepithema humile,* and *Formica pallidefulva* (Table S4)*.* Ants were absent throughout all observation months on non-infested crape myrtles.

**Fig. 1:**
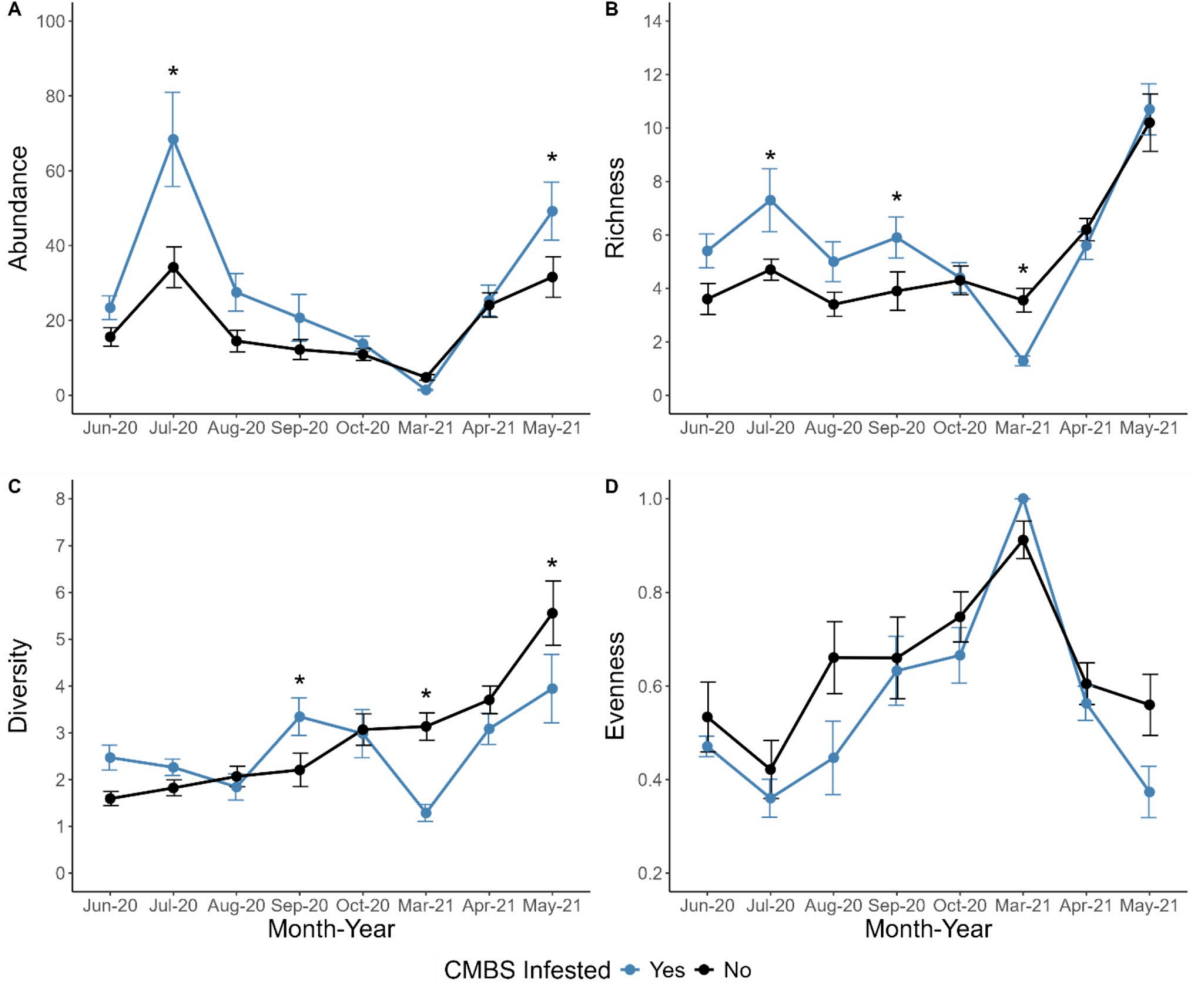
The seasonal effect of CMBS (*Acanthococcus lagerstroemiae*) infestation on **A** insect abundance, **B** family-level richness, **C** diversity, and **D** community evenness. Figures are plotted with standard errors. Asterisks indicate significant differences (*P* < 0.05) between infested and not infested trees based on least-squared differences (LSD) post hoc test, and the error bars are standard errors

Overall insect abundance was higher on CMBS-infested trees, but only in some months (Fig. 1A). Infestation by month interaction had a statistically significant effect on the abundance of insects on crape myrtle trees (LMM, *F* = 2.55, df = 7,122.8, *P* = 0.017). The interaction between treatment and month explained 49% of the variation in abundance, while site explained 3% of the variation in abundance (model r^2^). In July, infested trees had 100% greater abundance of insects relative to non-infested trees (*P* = 0.008; Fig. 1A). In May, infested trees had 56% greater abundance of insects relative to control trees (*P* = 0.015; Fig. 1A). In August, infested trees had 90% greater abundance of insects relative to non-infested trees (*P* = 0.071; Fig. 1A). All other comparisons were not statistically significant (*P* > 0.2).

Insect richness tended to be higher on CMBS-infested trees, but this varied across months (Fig. 1B). Infestation by month interaction had a statistically significant effect on family richness of insects on crape myrtle trees (*F* = 2.47, df = 7,122.7, *P* = 0.021, Table 1). The interaction between infestation and month explained 52% of the variation in family richness, while site explained 3% of the variation in family richness. In June, July, August, and September, richness was on average 51% greater on infested trees (Fig. 1B). These differences were most pronounced in July (55%, *P* = 0.0077) and September (51%, *P* = 0.039), but with similar trends in June (50%, *P* = 0.063) and August (47%, *P* = 0.098). However, in March we observed the opposite pattern, where infested trees had a 61% decrease in family richness relative to non-infested trees (*P* = 0.053, Fig. 1B). All other comparisons were not statistically significant (*P* > 0.5).

**Table 1:** The effects of CMBS infestation, month, and their interaction on measurements of insect diversity on crape myrtle trees

	Treatment				Month			Treatment × month		
	DF	*F*	*P*	Effect size	DF	*F*	*P*	DF	*F*	*P*
Abundance	1,9	17.46	0.002	+ 60%	7,139	17.49	< 0.0001	7,139	2.56	0.017
Richness	1, 9	4.87	0.055	+ 17%	7,122.7	22.71	< 0.0001	7,122.7	2.47	0.021
Diversity	1,9	1.04	0.33	-6%	7, 122.6	13.37	< 0.0001	7, 122.6	4.0	< 0.0001
Evenness	1,9	7.07	0.026	-14%	7,122.7	15.19	< 0.0001	7,122.7	1.15	0.33

The effect size for treatment is the overall effect of infestation on the variable relative to the non-infested group

In some months, diversity was higher on CMBS-infested trees, while in others it was higher on non-infested trees (Fig. 1C). Infestation by month interaction had a statistically significant effect on family richness of insects on crape myrtle trees (*F* = 4.01, df = 7,122.6, *P* < 0.001, Table 1). The interaction between treatment and month explained 43% of the variation in insect diversity, while site explained 0.7% of the variation in insect diversity. In September, infested trees had a 52% increase in insect diversity relative to non-infested trees (*P* = 0.03; Fig. 1C). There was also a similar trend in June with infested trees having a 55% increase in insect diversity (*P* = 0.09). However, March and May had the opposite pattern (Fig. 1C). In March, infested trees had a 59% decrease in insect diversity relative to the control trees (*P* = 0.003) and in May that difference was 29% (*P* = 0.002). All other comparisons were not statistically significant (*P* > 0.35).

Insect community evenness was affected by infestation (LMM, *F* = 7.07, df = 1,8.97, *P* = 0.026) and month (*F* = 15.19, df = 7, 122.73, *P* < 0.0001), but not their interaction (*F* = 1.15, df = 7,122.73 *P* = 0.33, Table 1). Infestation explained 3% of the variation in community evenness, month explained 39% of the variation in community evenness, and site explained 3% of the variation in community evenness. Infested trees had a 14% decrease in community evenness relative to non-infested trees (*P* = 0.034; Fig. 1D). In March, insect evenness was at least 35% greater than in other months and statistically differed from all months (*P* ≤ 0.0026; Fig. 1D). Insect community evenness in October was 41% greater than in June (*P* = 0.014), 81% greater than in July (*P* < 0.0001), and 52% greater than in May (*P* = 0.0017). Insect community evenness in September was 65% greater than in July (*P* = 0.0006). Insect community evenness in September was 39% greater than in May; however, this was not statistically significant (*P* = 0.05). Insect community evenness in April was 49% greater than in July (*P* = 0.026). All other comparisons were not statistically significant (*P* > 0.20).

**Fig. 2:**
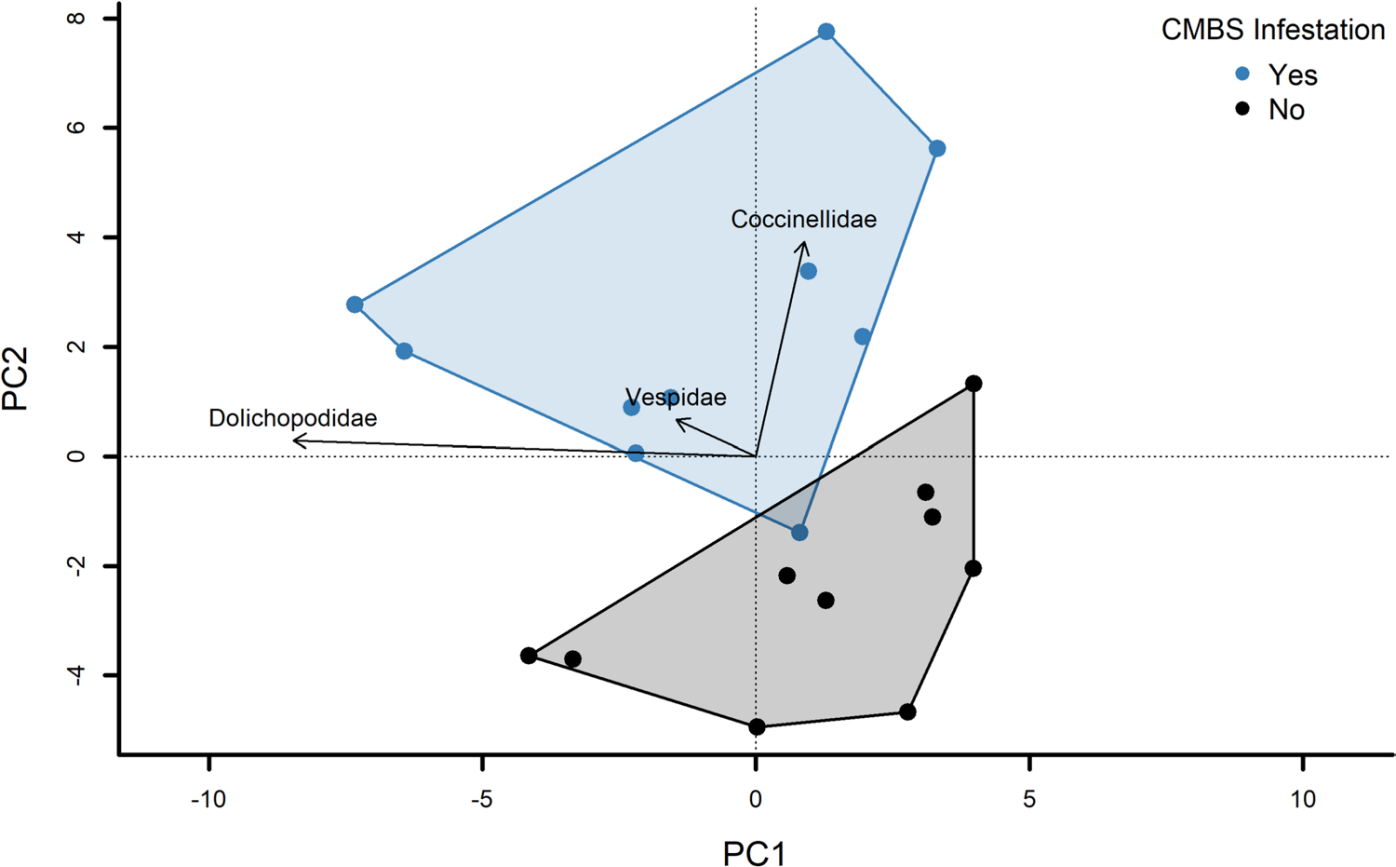
The effects of CMBS infestation on local insect community structure on crape myrtle trees. Data is visualized using principal component analysis. Vectors represent insect families

Overall community composition was distinct between infested and non-infested crape myrtle trees, and infestation status explained 14.3% of the variation in community composition (perMANOVA, *F* = 2.99, df = 1,17, *P* < 0.02). Our PCA depicted Coccinellidae and Vespidae aligned along PC2, oriented along the treatment-structured variation and toward the infested tree PC scores (Fig. 2).

### Associations between insect taxa and infested crape myrtle trees and their seasonality

The overall indicator taxa on infested trees were Vespidae (multipattern analysis, IV = 0.943, *P* = 0.0011), Coccinellidae (IV = 0.832, *P* = 0.0028), Syrphidae (0.798; *P* = 0.046), Muscidae (IV = 0.783, *P* = 0.049), Sphecidae (IV = 0.74, *P* = 0.044), and Halictidae (IV = 0.71, *P* = 0.033). Of these taxa, Coccinellidae, Muscidae, and Vespidae were the most abundantly recorded indicator taxa during the experiment (> 100 individuals recorded; Table S3). In total, 69.3% of Coccinellidae, ~ 90% of Vespidae, and 61.3% of Muscidae were recorded on infested trees throughout the observation months (Table S3). Of the 41 Syrphidae recorded in the experiment, 71% were on infested trees (Table S3). Of the 12 Sphecidae recorded in the experiment, 92% were on infested trees (Table S3). A total of 12 Halictidae were recorded in the experiment exclusively from infested trees (Table S3). No taxa were found to be overall indicators of non-infested trees.

Four families were seasonally associated with trees infested with CMBS (multipattern analysis; Table 2). Coccinellidae was associated with infested trees in the months of June and May (Table 2). Of the 58 Coccinellids recorded in June, 88% were on infested trees. Of the 348 Coccinellidae recorded in May, 74% were on infested trees. Vespidae was associated with infested trees in the months of July and September (Table 2). Of the 112 Vespidae recorded in July, 95% were on infested trees. Of the 34 Vespidae recorded in September, 88% were on infested trees. Dolichopodidae was associated with infested trees in the month of August (Table 2). Of the 307 Dolichopodidae recorded in August, 68% were on infested trees. Muscidae was associated with infested trees in August (Table 2). Of the 26 Muscidae recorded in August, 85% were on infested trees.

**Table 2:** Insect families associated with infested and non-infested trees during each observation month. IV is the strength of association between insect families and infested or non-infested trees

Month	Infested (Y/N)	Family	IV	*P* value
June-20	Y	Coccinellidae	0.84	0.020
July-20	Y	Vespidae	0.87	0.014
August-20	Y	Muscidae	0.77	0.019
	Y	Dolichopodidae	0.83	0.033
September-20	Y	Vespidae	0.89	0.013
October-20	-	-	-	-
March-21	N	Muscidae	0.78	0.014
April-21	-	-	-	-
May-21	Y	Coccinellidae	0.86	0.019

One family showed a seasonal association with non-infested trees. Muscidae were significantly associated with non-infested trees in the month of March (Table 2). A total of seven Muscidae were recorded in March exclusively from non-infested trees. No other taxa were associated with non-infested trees in other observation months.

## Discussion

The results from our study support our hypotheses and point to two main conclusions: (1) insect biodiversity patterns on crape myrtles were affected by both CMBS infestation and seasonality, resulting in distinct community structures, and (2) these effects were partly driven by the formation of seasonal associations of scale insect predators and carbohydrate foragers. First, we found that insect abundance and richness were elevated on CMBS-infested trees, specifically during the warmer months of May–September. These increases were not seasonally uniform, with March showing the opposite pattern of non-infested trees having greater abundance and richness. In March, infested crape myrtles had not yet flushed their leaves, while non-infested trees had fully developed foliage, creating structural and microclimatic differences between treatments. Additionally, infested trees exhibited visible buildup of sooty mold. This suggests that the effects of resource inputs resulting from CMBS infestation on local insect communities may also interact with its effects on host plant phenology and other biotic factors.

Our findings suggest that the introduction of CMBS restructures community dynamics on crape myrtle trees by filling the role of primary consumer. As a non-native tree with few ecological interactions, crape myrtle acts as an ecological substrate, while CMBS acts as an ecological catalyst, initiating the formation of novel trophic links. Without CMBS infestation, crape myrtles remained largely unutilized by insects. When infested with CMBS, crape myrtles became concentrated sites of insect activity, particularly for natural enemies and carbohydrate scavengers. Similar dynamics have been observed in vertebrate systems, where introduced herbivores facilitate increased populations of predators and scavengers (Roemer et al. 2001; Gangoso et al. 2006). This suggests that when herbivores are introduced into novel environments, they can initiate community restructuring via increased resource availability to higher trophic levels.

Months where infested crape myrtles experienced large increases in insect abundance and species richness often came at the expense of community evenness, aligning with our second hypothesis that CMBS facilitates communities dominated by generalist or opportunistic taxa due to its escape from co-evolved enemies and mutualists. In the summer and early fall, CMBS-infested trees supported elevated abundance and richness, but the majority of the community was composed of one or two taxa. This uneven distribution suggests that CMBS catalyzes interactions with opportunistic, generalist taxa that are best able to exploit a wide breadth of carbohydrate resources such as honeydew or are generalist predators of small soft-bodied insects. As a result, rather than fostering a diverse and balanced assemblage, CMBS promotes a narrow suite of responsive species.

Importantly, the dominant taxa associated with CMBS-infested trees were often exotic and invasive. The predominance of *H. axyridis, Vespula germanica*, and *Solenopsis invicta* on infested trees illustrates how one exotic species can facilitate the success of others. This bolstering of invasive species may have unintended spillover effects for native insect fauna via the effects of hyperpredation, which has been observed in other systems (Roemer et al. 2001; O’Dowd et al. 2003; Zhou et al. 2012). For example, feral pigs introduced to California islands provided a reliable food source for golden eagles, which in turn increased in number and caused hyperpredation on native island foxes (Roemer et al. 2001). This outcome affirms our third and fourth predictions by demonstrating that community structure was not only statistically distinct but also ecologically different, with recurring dominance by a small number of responsive taxa shaping the overall assemblage patterns. These cascading interactions resemble previously described invasion-driven mutualisms between honeydew producers and generalist consumers (Beggs 2001; Styrsky and Eubanks 2007; Zhou et al. 2012) and reflect a “domino effect” in which the establishment of one invader creates ecological conditions favorable for the success of others.

The impacts of an exotic scale species on insect communities would be more likely to reduce biodiversity in cases where the host plant is in the native range. Native tree species have more specialized associations with native insects that could be altered after scale infestation through impacts associated with tree diebacks or displacement of native insects resulting from trophic cascade. For example, the introduction of pine scale (*Marchalina hellenica*) into Greece pinewoods resulted in multiple losses of site-associated insect species, while increasing wood-feeding beetle species that were attributed to scale-induced morbidity of native pine trees (Petrakis et al. 2011). In contrast to systems where scale insects reduce biodiversity, our study documented increases in abundance and richness. However, these increases largely benefited exotics and generalists, not native entomofauna. This may be because urban ornamental landscapes, like those centered around crape myrtle, are dominated by turnover of human-selected, often exotic plant species that lack co-evolutionary interactions with native fauna (Kowarik 2011; Wheeler et al. 2022). In other words, urban plant communities are frequently reset based on aesthetic preferences rather than shaped by ecological selection processes. As such, they create stable, predictable environments for invasion cascades. Our study reinforces the idea that in urban settings, invasive herbivores like CMBS can restructure entire communities by adding trophic links to previously inert habitats.

These insights reinforce the need to incorporate temporal resolution into studies of invasion ecology. Without monthly sampling, the associations we observed between CMBS and dominant taxa would have remained hidden. These findings highlight that fine-scale temporal sampling is essential for detecting context-dependent impacts of invasive species, revealing seasonal associations between CMBS and insect taxa that would otherwise go unnoticed and provide insight into how invasion effects may fluctuate across biologically meaningful periods.

Together, our findings emphasize the role of invasive species not only as direct consumers but as catalysts that can structure ecological networks. CMBS creates novel resource flows that establish community structure on an exotic tree, in a context decoupled from evolutionary history. Understanding how such “ecological catalysts” operate is critical to predicting and mitigating the long-term ecological consequences of biological invasions of scale insects in human-modified environments.

## Supplementary Information

Below is the link to the electronic supplementary material.

Supplementary file1 (DOCX 164 KB)

Supplementary file2 (MPG 19312 KB)

Supplementary file3 (MPG 3900 KB)

Supplementary file4 (MPG 7152 KB)

## Data Availability

All data and source codes are available to access at GitHub: https://github.com/EPAC2328/CMBS_Commnty
