# Supplementary material for: Crape myrtle bark scale Acanthococcus lagerstroemiae (Coccidae: Eriococcidae) infestation seasonally alters the insect biodiversity on crape myrtle trees
Source: Oecologia. 2025 Sep 10;207(10):155. doi: 10.1007/s00442-025-05792-3 (PMC12423138; doi:10.1007/s00442-025-05792-3)
Supplement: Supplementary file 1 — Supplementary file1 (DOCX 164 KB) [file 442_2025_5792_MOESM1_ESM.docx]

Crape myrtle bark scale *Eriococcus lagerstroemiae* (Coccidae: Eriococcidae) infestation seasonally alters the abundance and composition of insect assemblages on crape myrtle trees

Elijah P. Carroll^1^, David W. Held ^1^*, Nash E. Turley^2^, Selina Bruckner^1^

^1^Department of Entomology and Plant Pathology, Auburn University, 301 Funchess Hall, Auburn, Alabama 36849 USA

^2^Department of Entomology, Pennsylvania State University, 501 Agricultural Sciences and Industries Building, State College, PA 16803

* Corresponding author: Elijah P. Carroll, [EPC0015@auburn.edu](mailto:EPC0015@auburn.edu)

**Supplementary methods**

Temperature and precipitation data collection

Temperature and precipitation data were obtained from Mesonet (<https://nationalmesonet.us/auburn/>). Data was recorded using a weather station located at Auburn University within 3 km from the experimental sites. Data on temperature was summarized for each collection date and each sampling time. Daily minimums, maximums, and averages are also presented. Data on precipitation was summarized as monthly averages.

Honeydew collection and analysis

Honeydew was collected during each two-day evaluation of insect visitation from the focal trees, weather permitting, to verify that honeydew was being produced during our observations. Because potted trees are soilless media and watered daily, they may not accurately represent seasonal honeydew production. To determine the seasonality of honeydew, collections were also made under the canopy of four crape myrtle trees established in the landscape between the months of June- October 2020 and March- May 2021. Trees near the Auburn University campus were scouted and four trees with infestations of CMBS and crape myrtle aphids were selected. The method for honeydew collection and extraction were adapted from Völkl et al. (1999). Paper dinner plates (Chinet, Desoto, KS, USA; 30 cm diam.) lined with plastic wrap (Press’n Seal, Glad, Oakland, CA, USA) were mounted onto a piece of PVC pipe with an inverted 2-liter soda bottle. The soda bottle was cut below the neck and affixed to the pipe so that the neck of the bottle fit over the PVC pipe leaving the cut open end to support the plate. The PVC pipe with the inverted bottle was hammered into the ground for stability under the dripline of the trees to be sampled. The plate was secured in place using metal wire (Galvanized Crafter's Wire, 19 Gauge, Hobby Lobby, Oklahoma City, OK, USA). Plates on the PVC stands were placed under the established trees for 48 hour each month between the months of June-Oct 2020 and March-May 2021.

After each collection, plates were brought back to the laboratory, and honeydew was quantified by washing each plate thoroughly with 30 mL of acetone. The solution was washed into 50 mL centrifuge tubes and dried under nitrogen for approx. 30 min to obtain the dry weight which was recorded. Dry weight was recorded as the difference between the weight of the clean 50 mL centrifuge tube before the acetone wash and the weight after drying under nitrogen. The honeydew mass collected from the potted infested trees in each pair under observation were not analyzed for seasonal differences.

Honeydew mass data collected from CMBS-infested landscape trees were fit to a linear regression. The model syntax was (honeydew ~ month). Means were separated using Tukey’s HSD (α< 0.05). The means and 95% confidence intervals of this data are presented (Supp. Figure 1B).

Descriptive statistics for biodiversity data

These data were obtained using the raw data of insects sampled from infested and non-infested trees from samples collected in June-Oct 2020 and March-May 2021. We included total abundances of each taxa collected during this experiment (Table S3), presence and absence and species of ants collected during each observation month (Table S4), abundances and species of Vespidae (Table S5), and Coccinellidae (Table S6).

**Supplementary Figures**


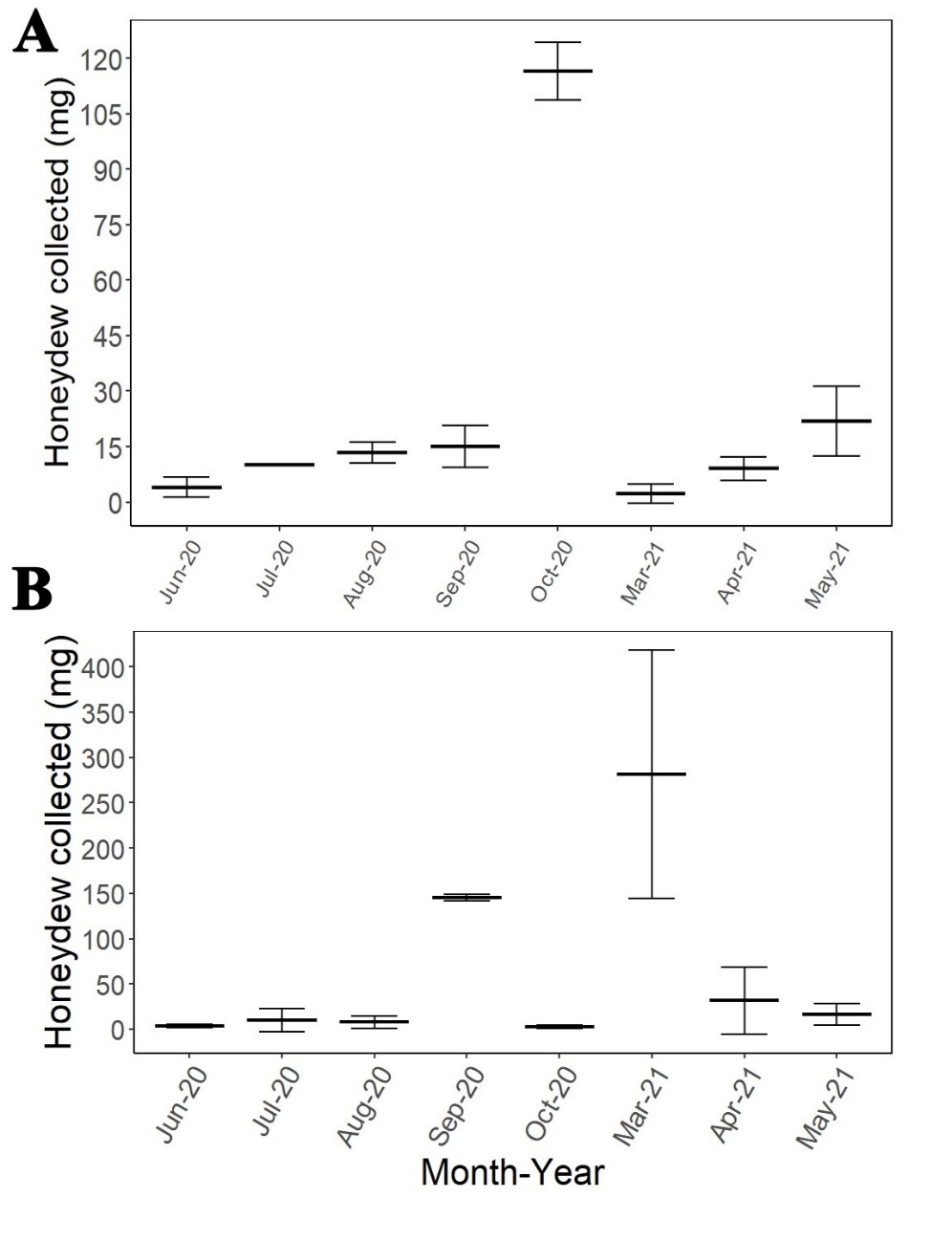


**Figure S1.** Mean dry weight of honeydew collected under A) landscape established, CMBS-infested crape myrtles and B) potted crape myrtles used for the visitation experiments during the months of July-Oct 2020 and March-May 2021. Figures are plotted with 95% confidence intervals.

On landscape crape myrtles, mean honeydew dry weight in October was significantly greater than that of all other months. Mean honeydew dry weight in May was significantly greater than honeydew dry weight recorded in April, June, and March (*P*< 0.05). Mean honeydew dry weight recorded in September was greater than that recorded in March (*P*<0.05) but did not differ from mean honeydew dry weight recorded in April, June, July, and August. Honeydew collections under infested potted trees confirmed the presence of honeydew coincident with months when trees were sampled for visitation (Figure S1B), ranging between 3.5 mg in October to 281.5 mg in March.

**Supplementary tables**

| **Table S1**. Temperature data for each collection date and each sampling time. | | | | | | | | |
| --- | --- | --- | --- | --- | --- | --- | --- | --- |
| Sampling date | Year | Air temperature (°F) at each sampling time | | | | Daily max (°F) | Daily min (°F) | Average (°F) |
|  |  | Daybreak | 9 AM | 12 PM | 3 PM |  |  |  |
| 29-June | 2020 | 73.3 | 76.9 | 86.1 | 91.2 | 91.4 | 70.4 | 79.4 |
| 30-June |  | 75.3 | 84.1 | 89.3 | 77 | 90.6 | 72.8 | 78.6 |
| 25-July |  | 75.2 | 82.3 | 88.1 | 91.3 | 91.7 | 71.6 | 79.6 |
| 26-July |  | 72.8 | 81 | 86.3 | 89.1 | 90.3 | 71.4 | 77.6 |
| 22-August |  | 69.2 | 78.3 | 83.1 | 85 | 85.4 | 68.5 | 77.31 |
| 23-August |  | 72.2 | 80.6 | 85.4 | 86 | 87 | 72 | 77.47 |
| 19-September |  | 69.5 | 70.3 | 71.4 | 71.2 | 71.9 | 65.1 | 69.2 |
| 20-September |  | 59.6 | 64.8 | 70.9 | 73.1 | 74.4 | 59.3 | 66.1 |
| 24-October |  | 69.5 | 71.8 | 71.2 | n/a | 73 | 67.4 | 69.4 |
| 25-October |  | 67.2 | 69.5 | 74.3 | 75.2 | 75.9 | 66.7 | 70.5 |
| 27-March | 2021 | 68.6 | 72.4 | 80.2 | 83.3 | 84.5 | 65.5 | 74 |
| 28-March |  | 66.9 | 74.6 | 67.9 | 65.3 | 75.9 | 55.3 | 66 |
| 29-April |  | 66.6 | 69.2 | 78.2 | 77.1 | 78.2 | 65.2 | 70.6 |
| 29-May |  | 68.2 | 73.2 | 76.7 | 77.7 | 78.2 | 60.1 | 70.7 |

| **Table S2**. Precipitation means for each sampling month. | | |
| --- | --- | --- |
| Month | Year | Mean monthly precipitation (mm/m^2^) |
| June | 2020 | 2.47 |
| July |  | 6.37 |
| August |  | 5.48 |
| September |  | 5.43 |
| October |  | 3.99 |
| March | 2021 | 6.26 |
| April |  | 3.09 |
| May |  | 1.09 |

| **Table S3.** The orders, families, and abundance of insects observed visiting crape myrtle trees, both infested and non-infested, over an eight-month period, excluding Formicidae. Infested and non-infested show the total abundances recorded on infested and non-infested trees, respectively. | | | | |
| --- | --- | --- | --- | --- |
| **Order** | **Family** | **Total abundance** | **Infested** | **Non-infested** |
| Coleoptera | Buprestidae | 13 | 4 | 9 |
|  | Cantharidae | 1 | 0 | 1 |
|  | Chrysomelidae | 16 | 9 | 7 |
|  | Coccinellidae | 604 | 419 | 186 |
|  | Curculionidae | 1 | 0 | 1 |
|  | Elateridae | 1 | 1 | 0 |
|  | Meloidae | 6 | 3 | 3 |
|  | Mordellidae | 14 | 9 | 5 |
|  | Cerambycidae | 1 | 1 | 0 |
|  | Scarabaeidae | 16 | 9 | 7 |
| Diptera | Agromyzidae | 7 | 5 | 2 |
|  | Anthomyidae | 2 | 2 | 0 |
|  | Asilidae | 7 | 3 | 4 |
|  | Bibionidae | 8 | 3 | 5 |
|  | Bombyliidae | 1 | 0 | 1 |
|  | Calliphoridae | 25 | 12 | 13 |
|  | Chamaemyiidae | 9 | 6 | 3 |
|  | Chironomidae | 58 | 23 | 35 |
|  | Chloropidae | 14 | 9 | 5 |
|  | Chyromyidae | 1 | 0 | 1 |
|  | Conopidae | 1 | 0 | 1 |
|  | Dolichopodidae | 1773 | 1031 | 742 |
|  | Drosophilidae | 42 | 39 | 3 |
|  | Empedidae | 2 | 1 | 1 |
|  | Lauxaniidae | 5 | 2 | 3 |
|  | Muscidae | 191 | 117 | 74 |
|  | Mycetophilidae | 5 | 2 | 3 |
|  | Phoridae | 226 | 116 | 110 |
|  | Piophilidae | 1 | 0 | 1 |
|  | Platystomatidae | 3 | 3 | 0 |
|  | Sarcophagidae | 82 | 52 | 30 |
|  | Sciaridae | 3 | 1 | 2 |
|  | Sciomyzidae | 3 | 0 | 3 |
|  | Syrphidae | 41 | 29 | 12 |
|  | Tabanidae | 2 | 1 | 1 |
|  | Tachinidae | 15 | 7 | 8 |
|  | Tephritidae | 9 | 8 | 1 |
|  | Therevidae | 4 | 1 | 3 |
|  | Tipulidae | 4 | 2 | 2 |
| Hemiptera | Anthocoridae | 3 | 3 | 0 |
|  | Cercopidae | 2 | 0 | 2 |
|  | Cicadellidae | 18 | 8 | 10 |
|  | Coreidae | 3 | 2 | 1 |
|  | Delphacidae | 2 | 1 | 1 |
|  | Flattidae | 1 | 1 | 0 |
|  | Membracidae | 7 | 4 | 3 |
|  | Pentatomidae | 6 | 2 | 4 |
|  | Plataspidae | 39 | 16 | 23 |
|  | Reduviidae | 7 | 3 | 4 |
| Hymentoptera | Apidae | 3 | 2 | 1 |
|  | Argidae | 1 | 0 | 1 |
|  | Braconidae | 16 | 10 | 6 |
|  | Chalcididae | 1 | 0 | 1 |
|  | Chrysididae | 2 | 2 | 0 |
|  | Crabronidae | 1 | 0 | 1 |
|  | Encyrtidae | 1 | 0 | 1 |
|  | Euchartidae | 0 | 0 | 0 |
|  | Eulophidae | 3 | 1 | 2 |
|  | Eupelmidae | 1 | 1 | 0 |
|  | Eurytomidae | 5 | 2 | 3 |
|  | Halictidae | 12 | 12 | 0 |
|  | Ichneumonidae | 180 | 98 | 82 |
|  | Mutillidae | 2 | 2 | 0 |
|  | Orussidae | 1 | 1 | 0 |
|  | Perilampidae | 1 | 0 | 1 |
|  | Pteromalidae | 3 | 2 | 1 |
|  | Scoliidae | 5 | 3 | 2 |
|  | Sphecidae | 12 | 11 | 1 |
|  | Tiphiidae | 15 | 10 | 5 |
|  | Vespidae | 181 | 161 | 20 |
| Neuroptera | Chrysopidae | 2 | 1 | 1 |
|  | Hemerobiidae | 2 | 0 | 2 |
| Psocodea | Psocidae | 11 | 4 | 7 |

| **Table S4.** Ant presence/absence data for each month and records to genus/species level. | | | |
| --- | --- | --- | --- |
| Month/year | (+/-) Infested | (+/-) Non-infested | Species |
| June, 2020 | + | - | *L. humile* |
| July, 2020 | + | - | *F. pallidefulva, S. invicta* |
| August, 2020 | + | - | *F. pallidefulva, S. invicta* |
| September, 2020 | + | - | *F. pallidefulva, S. invicta* |
| October, 2020 | + | - | *L. humile* |
| March, 2021 | - | - | - |
| April, 2021 | - | - | *-* |
| May, 2021 | + | - | *L. humile* |

| **Table S5.** Vespidae samples collected and identified to species-level from crape myrtle trees during the visitation experiment. Infested and non-infested are the abundances of species collected on CMBS infested and non-infested trees. | | | |
| --- | --- | --- | --- |
| Species | Total abundance | Infested | Non-infested |
| *Polistes exclamans* | 9 | 8 | 1 |
| *Polistes carolina* | 3 | 0 | 3 |
| *Polistes rugibinosus* | 2 | 1 | 0 |
| *Vespula squamosa* | 3 | 2 | 1 |
| *Vespula germanica* | 11 | 11 | 0 |
| *Vespula vulgaris* | 7 | 7 | 0 |
| No. species: 6 | Sample size= 34 | 29 | 5 |

| **Table S6.** Species/Genera and total abundances of ladybeetles visiting crape myrtles during the field surveys. Infested and non-infested are the abundances of species collected on CMBS infested and non-infested trees. | | | |
| --- | --- | --- | --- |
| Species | Total abundance | Infested | Non-infested |
| *Chilocorus stigma* | 17 | 10 | 7 |
| *Coccinella septempunctata* | 11 | 9 | 2 |
| *Coleomegilla maculata* | 7 | 3 | 4 |
| *Cycloneda munda* | 5 | 5 | 0 |
| *Harmonia axyridis* | 529 | 369 | 160 |
| *Hippodamia convergens* | 2 | 1 | 1 |
| *Hyperaspis bigeminata* | 7 | 2 | 5 |
| *Scymnus sp.* | 26 | 20 | 6 |
| No. genera: 8 | Total abundance= 604 | 419 | 185 |
